# Zebrafish Trak proteins 1a and 2 localize to the mitochondria

**DOI:** 10.17912/micropub.biology.000318

**Published:** 2020-10-16

**Authors:** Kelsey A Oonk, Lauren B Bienvenu, Paxton S Sickler, Christine Martin, Emily Nickoloff-Bybel, Albert Matthew Volk, Douglas C Weiser, Susan Walsh

**Affiliations:** 1 Department of Cell Biology, Duke University, Durham, NC 27710, USA; 2 Department of Biology, Rollins College, Winter Park, FL 32789, USA; 3 Department of Pharmacology and Physiology, Drexel University College of Medicine, Philadelphia, PA 19102, USA; 4 College of Medicine, University of Central Florida, Orlando, FL 32827, USA; 5 Department of Biological Sciences, University of the Pacific, Stockton, CA 95211, USA; 6 Life Sciences, Soka University of America, Aliso Viejo, CA 92656, USA

**Figure 1. The zebrafish Trak proteins are paralogous to each other, and EGFP-tagged Trak1a and 2 proteins localize to the mitochondria when expressed in mammalian tissue culture cells.**
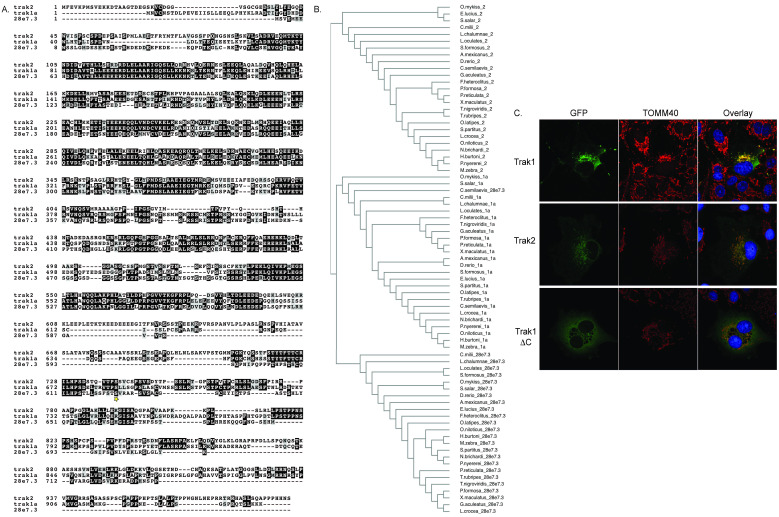
A. *D. rerio* sequences used for alignment: trak1a (GenBank XM_001921277.3), *si:dkey-28e7.3*-201 (ENSDARG00000074508), and trak2-201: (ENSDARG00000102471). The star represents the position of an intron in the cloned *trak1a* gene to generate a truncated protein (Trak1 ΔC). B. Various fish species also have three distinct genes which predominantly group with *trak1a, trak2,* and *si:dkey-28e7.3.* C. COS7 cells were transfected with zebrafish Trak1a/pEGFP, Trak2/pEGFP, or Trak1 ΔC/pEGFP plasmids using Lipofectamine 3000 (GFP). After expression, cells were fixed with methanol and immunostained to identify mitochondria using a human TOMM40 primary antibody and TRITC secondary antibody. Nuclei were counterstained with DAPI. Overlay is presented at far right. Images were collected at 630X magnification on a Zeiss LSM 700 confocal microscope, and representative images are displayed.

## Description

Inside a cell, mitochondria are organelles that exhibit dynamic locomotion and spatial rearrangement (Cai and Sheng 2009; Sheng 2017). This movement is necessary for a cell to maintain basic metabolic functions, and disruption of this motility often results in cell death. In fruit flies and mammals, one protein complex is primarily responsible for trafficking mitochondria along microtubules. This protein complex typically, but not always, consists of three proteins: Miro, Trak, and a motor protein (Stowers *et al.* 2002; Guo *et al.* 2005; Glater *et al.* 2006; Macaskill *et al.* 2009; Koutsopoulos *et al.* 2010; Brickley and Stephenson 2011; van Spronsen *et al.* 2013; Barel *et al.* 2017; López-Doménech *et al.* 2018; Henrichs *et al.* 2020).

In contrast to the single *Drosophila* protein Milton (Stowers *et al.* 2002), there are two mammalian genes of the Trak protein family: *trak1* and *trak2* (Koutsopoulos *et al.* 2010; Brickley and Stephenson 2011; van Spronsen *et al.* 2013). Both proteins have also been called huMilt1 and huMilt2, OIP106 and OIP98, or ALS2CR3/KIAA0549 and GRIF-1, respectively (Beck *et al.* 2002; Iyer *et al.* 2003; Brickley *et al.* 2005; Gilbert *et al.* 2006). Overexpression of either human Trak protein in mammalian cells generates abnormal clumping of the mitochondria, indicating that these proteins regulate mitochondrial motility and maintain the normal network of mitochondria in the cell (Koutsopoulos *et al.* 2010). In contrast, by reducing Trak protein levels in rat hippocampal neurons, Trak1 was identified as necessary for mitochondrial movement, yet Trak2 was not. However, Trak2 appears to be partially redundant in function with Trak1 since increasing Trak2 protein levels can rescue the loss of Trak1 protein (Brickley and Stephenson 2011). Discrepancy in the structure of the two paralogs may allow them to perform unique functions within the mitochondrial trafficking process (van Spronsen *et al.* 2013). For example, Trak1 associated with both the kinesin and dynein motor protein complex. In contrast, the Trak2 protein adopts a different structure that interferes with kinesin binding, only permitting interaction with dynein (van Spronsen *et al.* 2013; Loss and Stephenson 2015). In this way, these two similar proteins are distinct in their cellular functions, since Trak1/kinesin interactions mediate mitochondrial transport towards the axon, and the Trak2/dynein interactions cause mitochondrial transport toward the dendrites (van Spronsen *et al.* 2013; Loss and Stephenson 2017). Overall, mitochondrial trafficking and cell viability is highly sensitive to the concentration of Trak proteins (Stowers *et al.* 2002; Webber *et al.* 2008; Brickley and Stephenson 2011; Barel *et al.* 2017).

Assuming conserved function, our research sought to characterize the Trak proteins in zebrafish, *D. rerio*. Zebrafish are an excellent vertebrate model system. Their entire genome has been sequenced, annotated, and revised multiple times. They reproduce and develop quickly in comparison to other species, permitting observation of the roles of Trak proteins throughout developmental stages. Additionally, they are fertilized externally and are transparent, simplifying the ability to study Trak in live animals. In zebrafish, the most recent Ensembl genome assembly (April 2018) suggests that there are three paralogs of *trak*: *trak1a*, *trak2*, and *si:dkey-28e7.3* (Figure 1A). *trak1a* is located on chromosome 16; *trak2* is on chromosome 6; and *si:dkey-28e7.3* is on chromosome 11. The fact that there are more than two genes is consistent with whole genome duplication in the teleost fish lineage (Meyer and Schartl 1999; Taylor *et al.* 2001; Taylor 2003; Woods *et al.* 2005). After genome duplication, redundant genes were pseudogenized, resulting in three instead of four genes (Meyer and Schartl 1999; Taylor *et al.* 2001). Indeed, three putative Trak genes are found in twenty-five other sequenced fish species, consistent with this hypothesis (Figure 1B and Table 1).

Based on findings about Trak in mammals and fruit flies, we aimed to understand this zebrafish protein family. As a first step, we co-localized each of the zebrafish Trak proteins with mitochondria in a heterologous system by cloning and overexpressing EGFP-tagged Trak proteins in easy-to-transfect and image mammalian tissue culture cells. *trak1a* and *trak2* were amplified from cDNA from pooled embryos (1, 2, and 5 days post fertilization (dpf)). *trak2* transcripts were expected to be abundant in this sample, based on high-throughput Expression Atlas data (Busch-Nentwich lab); there are no current expression data for either *trak1a* or *si:dkey-28e7.3*. For primers to *trak1a,* we used the original gene sequence from GenBank (XM_001921277.3), which has a longer N-terminus than the most recent version of the *trak1a* gene from Ensembl; a starting ATG occurs 225 nucleotides upstream of the ATG noted in *trak1a-202* (atga to acag)*.* We could not amplify an intact *si:dkey-28e7.3* transcript, suggesting it might not be expressed or that the transcript may be of low abundance at these developmental time points. The *trak1a* and *trak2* genes were cloned into pEGFP vectors and transfected into COS7 cells, and cells were immunostained for the endogenous outer mitochondrial protein TOMM40 (Figure 1C). In both cases, overexpressed Trak1a- and Trak2-EGFP proteins co-localized with TOMM40 and caused mitochondrial clumping, like their mammalian orthologs (Koutsopoulos *et al.* 2010). Notably, we also cloned a *trak1a* gene that had an intron at position 686 (starred in Figure 1A) that caused a frameshift nonsense mutation, resulting in a truncated C-terminus (Trak1 ΔC). This aberrant protein was predominantly cytosolic, rather than mitochondrial (Figure 1C), suggesting that the C-terminus is important for appropriate mitochondrial localization. These data are consistent with data regarding the human Trak1 protein where a protein containing only amino acids 1-734 (of 953) was cytosolic instead of mitochondrial (Koutsopoulos *et al.* 2010). Although we could not obtain a full-length *si:dkey-28e7.3* transcript to test in our system, we predict that *si:dkey-28e7.3* is unlikely to localize to the mitochondria, given that we demonstrate that the C-terminus of *trak1a* seems to anchor the protein to the mitochondria and this region is the most divergent in *si:dkey-28e7.3* (Figure 1A)*. si:dkey-28e7.3* is also less similar to the mammalian orthologs that align well with *D. rerio trak1a* and *trak2.*

Using the preliminary data generated from this project, we hope that the zebrafish Trak protein family can be further analyzed *in vivo*, allowing for better understanding of how Trak proteins contribute to mitochondrial movement in a live vertebrate animal. A previous study in zebrafish did not report a phenotype after injection of a splicing morpholino targeted to *trak1* (Choksi *et al.* 2014). This result is not surprising given data from the knockdown of Miro where a morphant phenotype is only observed when all three paralogs are depleted, suggesting redundant functions (Hollister *et al.* 2016). CRISPR technology utilized in the context of zebrafish expressing mitochondrial fluorescent proteins may be a means to stably generate double and triple mutants to measure the effects at a cellular and organismal level (Fichi *et al.* 2019; Arribat *et al.* 2019). Greater knowledge of these motility mechanisms may eventually be extrapolated to neurodegenerative diseases, such as ALS and spastic paraplegia, where mitochondrial trafficking plays a significant role.

## Methods

**Phylogenetics**

Alignments were created from Ensembl sequences from zebrafish GRCz11 (Yates *et al.* 2020) for *si:dkey-28e7.3*-201 (ENSDARG00000074508) and *trak2-201* (ENSDARG00000102471) and GenBank for *trak1a* (XM_001921277.3; https://www.ncbi.nlm.nih.gov/nuccore/XM_001921277.3). Sequences were inputted into Clustal Omega (https://www.ebi.ac.uk/Tools/msa/clustalo/; Madeira *et al.* 2019) and formatted using BoxShade (https://embnet.vital-it.ch/software/BOX_form.html) or Guide Tree Cladogram to show the relationships among the various species. Sequence data from other fish species were compiled through examining various fish genomes available on Ensembl and utilizing Ensembl’s BLAST/BLAT tool to search for Trak orthologs. A complete list of the sequence files is in Table 1.

**Table 1. Sequences used for the cladogram in Figure 1B.**

**Table:** 

species	transcipt name	gene ID	assembly
Astyanax mexicanus	si:dkey-28e7.3-201	ENSAMXG00000018307	Astyanax_mexicanus-2.0 (September 2017)
Astyanax mexicanus	trak1a-201	ENSAMXG00000029426	Astyanax_mexicanus-2.0 (September 2017)
Astyanax mexicanus	trak2-201	ENSAMXG00000012437	Astyanax_mexicanus-2.0 (September 2017)
Callorhinchus milii	si:dkey-28e7.3-202	ENSCMIG00000017409	Callorhinchus_milii-6.1.3 (December 2013)
Callorhinchus milii	trak2-202	ENSCMIG00000009046	Callorhinchus_milii-6.1.3 (December 2013)
Callorhinchus milii	trak1a-202	ENSCMIG00000006222	Callorhinchus_milii-6.1.3 (December 2013)
Cynoglossus semilaevis	Unnamed (listed in tree as C.semilaevis_28e7.3)	ENSCSEG00000006363	Cse_v1.0 (January 2014)
Cynoglossus semilaevis	trak2-201	ENSCSEG00000014003	Cse_v1.0 (January 2014)
Cynoglossus semilaevis	trak1a-201	ENSCSEG00000003758	Cse_v1.0 (January 2014)
Danio rerio	si:dkey-28e7.3-201	ENSDARG00000074508	GRCz11 (May 2017)
Danio rerio	trak2-201	ENSDARG00000102471	GRCz11 (May 2017)
Danio rerio	trak1a-202	ENSDARG00000041304	GRCz11 (May 2017)
Esox lucius	si:dkey-28e7.3-201	ENSELUG00000009890	Eluc_v4 (April 2019)
Esox lucius	trak2-202	ENSELUG00000002196	Eluc_v4 (April 2019)
Esox lucius	trak1a-204	ENSELUG00000004615	Eluc_v4 (April 2019)
Fundulus heteroclitus	si:dkey-28e7.3-202	ENSFHEG00000001760	Fundulus_heteroclitus-3.0.2 (January 2015)
Fundulus heteroclitus	trak2-201	ENSFHEG00000007156	Fundulus_heteroclitus-3.0.2 (January 2015)
Fundulus heteroclitus	trak1a-201	ENSFHEG00000016654	Fundulus_heteroclitus-3.0.2 (January 2015)
Gasterosteus aculeatus	si:dkey-28e7.3-201	ENSGACG00000018327	BROAD S1 (February 2006)
Gasterosteus aculeatus	trak1a-201	ENSGACG00000006003	BROAD S1 (February 2006)
Gasterosteus aculeatus	trak2-201	ENSGACG00000014156	BROAD S1 (February 2006)
Haplochromis burtoni	si:dkey-28e7.3-201	ENSHBUG00000000586	AstBur1.0 (December 2011)
Haplochromis burtoni	trak1a-203	ENSHBUG00000011007	AstBur1.0 (December 2011)
Haplochromis burtoni	trak2-202	ENSHBUG00000016106	AstBur1.0 (December 2011)
Larimichthys crocea	si:dkey-28e7.3-202	ENSLCRG00005003733	L_crocea_2.0 (November 2018)
Larimichthys crocea	trak2-203	ENSLCRG00005003901	L_crocea_2.0 (November 2018)
Larimichthys crocea	trak1a-204	ENSLCRG00005020918	L_crocea_2.0 (November 2018)
Latimeria chalumnae	si:dkey-28e7.3-201	ENSLACG00000007300	LatCha1 (September 2011)
Latimeria chalumnae	trak2-201	ENSLACG00000006273	LatCha1 (September 2011)
Latimeria chalumnae	trak1-201	ENSLACG00000001844	LatCha1 (September 2011)
Lepisosteus oculates	si:dkey-28e7.3-201	ENSLOCG00000013653	LepOcu1 (December 2011)
Lepisosteus oculates	trak1a-201	ENSLOCG00000001329	LepOcu1 (December 2011)
Lepisosteus oculates	trak2-201	ENSLOCG00000010723	LepOcu1 (December 2011)
Maylandia zebra	si:dkey-28e7.3-201	ENSMZEG00005008443	M_zebra_UMD2a (April 2018)
Maylandia zebra	trak2-201	ENSMZEG00005006192	M_zebra_UMD2a (April 2018)
Maylandia zebra	trak1a-202	ENSMZEG00005002331	M_zebra_UMD2a (April 2018)
Neolamprologus brichardi	si:dkey-28e7.3-201	ENSNBRG00000023010	NeoBri1.0 (December 2011)
Neolamprologus brichardi	Unnamed (listed in tree as N.brichardi_1a)	ENSNBRG00000010142	NeoBri1.0 (December 2011)
Neolamprologus brichardi	trak2-201	ENSNBRG00000014231	NeoBri1.0 (December 2011)
Oncorhynchus mykiss	si:dkey-28e7.3-205	ENSOMYG00000018119	Omyk_1.0 (June 2017)
Oncorhynchus mykiss	Unnamed (listed in tree as O.mykiss_1a)	ENSOMYG00000039092	Omyk_1.0 (June 2017)
Oncorhynchus mykiss	trak2-201	ENSOMYG00000011517	Omyk_1.0 (June 2017)
Oreochromis niloticus	si:dkey-28e7.3-205	ENSONIG00000018805	O_niloticus_UMD_NMBU (June 2018)
Oreochromis niloticus	trak1a-202	ENSONIG00000007240	O_niloticus_UMD_NMBU (June 2018)
Oreochromis niloticus	trak2-202	ENSONIG00000011992	O_niloticus_UMD_NMBU (June 2018)
Oryzias latipes	si:dkey-28e7.3-203	ENSORLG00000025102	ASM223467v1 (July 2017)
Oryzias latipes	trak1a-201	ENSORLG00000005943	ASM223467v1 (July 2017)
Oryzias latipes	trak2-201	ENSORLG00000024460	ASM223467v1 (July 2017)
Poecilia formosa	si:dkey-28e7.3-201	ENSPFOG00000003564	Poecilia_formosa-5.1.2 (October 2013)
Poecilia formosa	trak1a-201	ENSPFOG00000018652	Poecilia_formosa-5.1.2 (October 2013)
Poecilia formosa	trak2-201	ENSPFOG00000001467	Poecilia_formosa-5.1.2 (October 2013)
Poecilia reticulata	si:dkey-28e7.3-203	ENSPREG00000000453	Guppy_female_1.0_MT (April 2014)
Poecilia reticulata	trak1a-204	ENSPREG00000013360	Guppy_female_1.0_MT (April 2014)
Poecilia reticulata	trak2-201	ENSPREG00000007459	Guppy_female_1.0_MT (April 2014)
Pundamilia nyererei	si:dkey-28e7.3-201	ENSPNYG00000002974	PunNye1.0 (December 2011)
Pundamilia nyererei	trak2-201	ENSPNYG00000009328	PunNye1.0 (December 2011)
Pundamilia nyererei	trak1a-201	ENSPNYG00000011722	PunNye1.0 (December 2011)
Salmo salar	si:dkey-28e7.3-201	ENSSSAG00000044165	ICSASG_v2 (June 2015)
Salmo salar	trak2-201	ENSSSAG00000031517	ICSASG_v2 (June 2015)
Salmo salar	trak1a-201	ENSSSAG00000004358	ICSASG_v2 (June 2015)
Scleropages formosus	si:dkey-28e7.3-201	ENSSFOG00015002821	fSclFor1.1 (April 2019)
Scleropages formosus	trak1a-208	ENSSFOG00015001806	fSclFor1.1 (April 2019)
Scleropages formosus	trak2-201	ENSSFOG00015004411	fSclFor1.1 (April 2019)
Stegastes partitus	si:dkey-28e7.3-201	ENSSPAG00000018438	Stegastes_partitus-1.0.2 (May 2014)
Stegastes partitus	trak1a-202	ENSSPAG00000017135	Stegastes_partitus-1.0.2 (May 2014)
Stegastes partitus	trak2-201	ENSSPAG00000001720	Stegastes_partitus-1.0.2 (May 2014)
Takifugu rubripes	si:dkey-28e7.3-201	ENSTRUG00000003718	fTakRub1.2 (June 2019)
Takifugu rubripes	trak2-201	ENSTRUG00000010804	fTakRub1.2 (June 2019)
Takifugu rubripes	trak1a-202	ENSTRUG00000002103	fTakRub1.2 (June 2019)
Tetraodon nigroviridis	si:dkey-28e7.3-201	ENSTNIG00000004864	TETRAODON 8.0 (March 2007)
Tetraodon nigroviridis	trak2-201	ENSTNIG00000010353	TETRAODON 8.0 (March 2007)
Tetraodon nigroviridis	trak1a-201	ENSTNIG00000006230	TETRAODON 8.0 (March 2007)
Xiphophorus maculatus	si:dkey-28e7.3-202	ENSXMAG00000000197	X_maculatus-5.0-male (December 2017)
Xiphophorus maculatus	trak1a-202	ENSXMAG00000008737	X_maculatus-5.0-male (December 2017)
Xiphophorus maculatus	trak2-201	ENSXMAG00000008010	X_maculatus-5.0-male (December 2017)

**Cloning and Expression**Total RNA was extracted from one, two, or five days post fertilization (dpf) wild-type AB zebrafish embryos and pooled. The embryos were homogenized in Trizol (Invitrogen) according to manufacturer’s directions. First strand cDNA synthesis was then carried out using 2μg of RNA and the Superscript III kit (Invitrogen). For *trak1a,* PCR was performed using Phusion HF DNA polymerase (NEB) and primers designed to GenBank XM_001921277.3 (zfTrak1.ECORI.F: 5’-GCCGAATTCATGAATGTGTGTAACAGCAC; zfTrak1.XHOI.R: 5’-CCGCTCGAGTCACTTTTTCTTGAGGC) to clone into pcGlobin2 (Ro *et al.* 2004). EcoRI and XbaI were then used to move the gene into pEGFP-C2 (Clontech). *trak2* was cloned directly into pEGFP-C2 using primers (zfTRAK2.XhoI.F: 5’-CGATCTCGAGCATGTTCGAGGTGAAGCC; zfTRAK2.XmaI.R: 5’-TGGGCCCTTATGAATTATGATGTGGGG) and the restriction enzymes XhoI and XmaI. Cloned genes were sequenced through Eurofins Genomics and compared to Genbank and Ensembl sequences using primers to the vector and internal primers (Trak1.811.F: 5’- GCACTTGAAAATGAAGAG; Trak1.1798.F: 5’-GTCGTGACCAAGGGC; TRAK1.2015.R: 5′-GCTCATCTGAAGGGTG; zfTRAK2.800.F: 5’-CTCCCAGAAGAATGAGGA; and zfTRAK2.1660.R.seq: 5’-TGGTGAAGGTGTAGGTG).

COS7 cells (ATCC) were cultured in Dulbecco’s Modified Eagle’s Medium containing 10% fetal bovine serum in a 37ºC incubator at 5% CO_2_. Confluent cells were split onto uncoated glass coverslips in a 6-well plate for transfection. COS7 cells were transfected with zebrafish Trak1/pEGFP-C2 or Trak2/pEGFP-C2, human Milton1/pEGFP or Milton2/pEGFP (provided by M.T. Ryan), or pEGFP-C2 using Lipofectamine 3000 (Invitrogen) following the manufacturer’s protocol. Proteins were expressed for 12 to 24 hours before processing for immunofluorescence. Transfected COS7 cells were washed in 1X PBS and fixed with 100% ice-cold methanol at -20°C for at least 10 minutes. The cells were washed three times for 5 minutes in 1X phosphate buffered saline (PBS). Fixed cells were blocked for 1 hour at room temperature on a rocker in 5% normal goat serum/0.3% Triton X/1X PBS. Rabbit polyclonal primary antibody to human TOMM40 (1:500; ULAB4; gift of C. M. Koehler) was added to blocking buffer and incubated at 4ºC overnight. The cells were washed 3 times with 1X PBS for 5 minutes and incubated with the secondary antibody goat-anti-rabbit-TRITC (1:1000; Jackson ImmunoChemicals) with 0.3% Triton X/1X PBS for 1 hour on a rocker. The cells were stained with DAPI/0.3% Triton X/1X PBS at room temperature for 5 minutes. The cells were washed twice with 1X PBS for 5 minutes and placed onto slides with Fluoromount-G (Southern Biotech). Images were collected using 630X magnification on a Zeiss LSM 700 confocal microscope. Transfection and imaging of constructs and their comparison to human Traks and untagged EGFP were performed for more than ten replicates, and representative images are shown.
